# Sealing Ability Promoted by Three Different Endodontic Sealers

**Published:** 2011-05-15

**Authors:** Graziele Silva, Emmanuel João Nogueira Leal da Silva, Juliana Melo da Silva, Carlos Vieira Andrade-Júnior, Caio César Randi Ferraz

**Affiliations:** 1. State University of Southwest Bahia, Bahia, Brazil.; 2. Piracicaba Dental School, State University of Campinas, Campinas, Brazil.; 3. Federal University of Pará, Pará, Brazil.

**Keywords:** Clearing, Endodontics, Microleakage, Root Canal Filling, Resilon, Sealer

## Abstract

**INTRODUCTION:**

The aim of this study was to evaluate the apical and coronal seal of endodontic filling promoted by the combined use of Sealer 26/gutta-percha, Endofill/gutta-percha and Resilon/ Epiphany.

**MATERIALS AND METHODS:**

A total of 38 extracted human canine teeth were selected; 10 teeth for each test group and 4 for each control group. After conducting conventional endodontic treatment, the teeth were immersed in saline solution for thirty days, and subsequently sealed and stored in India ink for seven days. They were then cleaned and evaluated for infiltration by stereoscopic microscope. The data were statistically analyzed by ANOVA and Bonferroni tests.

**RESULTS:**

The results showed no significant differences between the three groups of filling materials used.

**CONCLUSION:**

Within the limitations of this in vitro study, no material showed superiority in their apical and coronal seal.

## INTRODUCTION

The root canal obturation is the final stage of the endodontic treatment [1]. Though all the stages of endodontic therapy have equal importance; more emphasis is given to obturation as its quality can determine the success or failure of therapy [2]. Moreover, the quality of the filling is used as parameter for assessing the quality of endodontic treatment.

A good root canal filling should be able to prevent fluids, residues of necrotic tissue, bacteria and other irritants that were not removed during the biomechanical preparation to be disseminate to the periapical region [2][3].

Different techniques and materials have been developed to obtain a hermetic sealing [2], however an ideal material has not been discovered yet [1][4][5]. Among the materials currently used, gutta-percha is known to have good physical and biological properties, however it also have unfavorable characteristics such as low flow and low adhesion [4][6]. Therefore, the use of sealers alongside gutta-percha produces a more homogeneous filling and prevents micro-leakage of periapical exudates, preventing reinfection [1][4]. This material is critical for hermetic seal since the infiltration of fluids into the filling may occur at the interfaces of the sealer with dentin surface, sealer with gutta-percha, inside the sealer or by the dissolution of the sealer [1][4][7].

Many investigations were carried out into sealers with physicochemical properties that can eliminate the possibility of recontamination and micro-leakage, and thus new materials were developed. These materials are classified according to their constituents, such as zinc oxide and eugenol, calcium hydroxide, resins, glass ionomer or silicones [1].

The aim of this study was to analyze the quality of apical and coronary seal promoted by the use of Sealer 26/gutta-percha, Endofill/gutta-percha and Resilon/Epiphany.

## MATERIALS AND METHODS

A total of 38 canines were selected from a teeth bank. After removal of the coronal portion, #10 K files (Dentsply, Maillefer, Baillaigues, Switzerland) were introduced into the canal until it was just visible at the apical foramen. From this length, 1 mm was subtracted to establish the working length (WL).

The root canals were prepared with K-Flexofile (Maillefer, Ballaigues, Switzerland), with the crown-down technique until WL was reached. Instruments were adjusted to the walls of the canal, followed by instruments in ascending order of diameter, to achieve an apical flare equal to that provided by #45 file. During the biomechanical preparation, patency was maintained with a size #10 K file and irrigation with 2.5% sodium hypochlorite was carried out. After preparation, the canals were instrumented with #10 and #15 files 2mm beyond the foramen, to standardize the foramina diameter. After using the last file, the roots were irrigated with physiologic saline solution and dried with absorbent paper cones (Tanari, Amazonas-Brazil).

To remove the smear layer, 17% EDTA (Biodynamic Quim, Paraná, Brazil), was mixed within the canal for 0.5 minutes with a lentulo spiral (Maillefer, Ballaigues, Switzerland), and then left for another 2.5 minutes in the canal. Sodium hypochlorite (2.5%) was subsequently applied under the same conditions as EDTA; however, this material remained for 4.5 minutes within the canal. The canal was irritated with physiologic saline solution and dried with absorbent paper cones.

Samples were divided into three experimental groups (n=10) and two control groups (n=4). The canals of group 1, 2 and 3 were filled with Sealer 26/gutta-percha, Endofill/gutta-percha and Resilon/Epiphany system, respectively. The tooth roots in group 4 were not filled and the coronal third was filled with Coltosol (Vigodent, Rio de Janeiro, Brazil) to facilitate sealing; the roots’ surfaces were then thoroughly coated with two layers of nail varnish (Colorama, São Paulo, Brazil). The root canals in group 5 were not filled, but received two coats of nail varnish except the apical 1mm and the cervical portion. The same protocol of coating was also used for the groups 1, 2 and 3.

Tagger´s hybrid technique was performed for root canal obturation in which gutta-percha was used with Endofill (Dentsply, Rio de Janeiro, Brazil) or Sealer 26 (Dentsply, Rio de Janeiro, Brazil). In the Resilon/Epiphany system (Pentron Clinical Technologies, Wallingford, CT, USA) obturation was performed according to the manufacturer using Tagger’s hybrid technique. A halogen light curing unit (Gnatus, São Paulo, Brazil) was used for 40 seconds to cure the coronal portion of Resilon/Epiphany group. Finally, the teeth were immersed in physiologic saline solution for 30 days. The solution was renewed each week. Later, the roots were covered with two coats of nail varnish as described above and were stored in India ink (Acrilex, São Paulo, Brazil) for seven days.

The teeth in groups 4 and 5 were also placed in the India ink for the same period. Specimens were washed with water and immersed in water for two hours. Once cleaning was performed, the apical and coronal leakage was analyzed through a stereomicroscope (Leica, Germany) with ×20 magnification. All teeth were observed by one calibrated evaluator and data were analyzed with ANOVA and Bonferroni tests (P<0.05) using the Biostat software.

## RESULTS

Apical leakage (Table 1) and coronal leakage (Table 2) showed no statistically significant differences between the three groups of materials. However, Sealer 26/Gutta-percha showed significantly higher apical leakage than the negative controls (P<0.05). All test groups showed significantly greater coronal microleakage than the negative controls (P<0.05).

**Table 1:** Apical microleakage of filling materials (mm)

**Filling Material**	**Mean ± SD **
**Sealer 26/gutta-percha**	2.67±2.50
**Endofill/gutta-percha**	1.10±0.66
**Resilon/Epiphany**	1.44±1.07
**Negative Control**	0.00±0.00
**Positive Control**	12.13±5.12

**Table 2:** Coronal microleakage of filling materials (mm)

**Filling Material**	**Mean±SD**
**Sealer 26/guta-percha**	3.94±3.57
**Endofill/guta-percha**	4.20±3.91
**Resilon/Epiphany**	2.44±1.48
**Negative Control**	0.00±0.00
**Positive Control**	13.50±2.41

## DISCUSSION

The quality of obturation seal has been a hot debate; especially the sealers used [1][2][3][4][8][9]. Conflicting results have been presented when different materials and methodologies were used. We used the dye penetration method to compare the quality of apical and coronal seal between the different filling systems. Various types of dyes and techniques such as India ink, methylene blue, bacterial penetration and filtration of fluids are used to evaluate the sealing ability of filling materials, however, as the dyes have smaller molecules than the pathogens that they intend to simulate, dyes application may have questionable validity [10]. In contrast to this finding, a review of a large number of publications on fluid filtration showed that the penetration of particles or solutions between the filling material and root canal walls are adequate measures to assess whether the root canal filling has adequate seal [11].

The chosen method for the evaluation of the filling was provided by the clearing technique. This has the advantage of easy implementation, economics, possibility of three-dimensional visualization of the teeth, reproducibility and reliability in the search results [6][7][12][13].

No statistical difference in microleakage was observed between the study groups; however, Sealer 26/gutta-percha showed statistical difference compared with the mean of negative controls; this could suggest the poorest seal for this filling system. Sealer 26 is an epoxy resin sealer with calcium hydroxide content [14]. This sealer has more biocompatibility and sealing than zinc oxide and eugenol sealers. However a study claimed that calcium hydroxide sealers present similar values of infiltration to other commonly used sealers; corroborating the findings of this study [15]. Using calcium hydroxide sealers is due to antimicrobial effects and stimulating periapical tissues to maintain or promote health. The sealing ability can be changed by the solubility of sealers. A desirable property of sealers is insolubility and constancy against action of tissue fluids. This is a major concern with calcium hydroxide sealer and its therapeutic effects [16].

In relation to coronal leakage, there were no significant differences between the various fillings, except the Resilon/Epiphany system which did not significantly differ from negative controls. The formation of a monobloc between the Resilon cones, Epiphany Sealer and the root canal walls, according to several studies [3][17][18][19], was not demonstrated in this study.

Endofill is a widely known and clinically used endodontic sealer. It is zinc-oxide and eugenol based and was used as a control in several studies that compare sealers in vitro [3]. There are conflicting results regarding the sealing ability of fillings with gutta-percha and zinc oxide and eugenol sealer [3][5]. It has been claimed that sealers containing eugenol can generate cause the volume expansion of gutta-percha and improved sealing [20]. No superior sealing was observed by Endofill in this study. Zinc oxide and eugenol based cements are highly disintegrable and soluble when kept in aqueous medium and are highly permeable, with poor adhesion to the dental structures [3][21]; this fact could not be seen in this study. Storing specimens in saline solution for 30 days may have increased the penetration of India ink, similar to Brandão et al.’s study [13].

The difficulty of linking the in vitro results to the clinical conditions and using variable methods may create the inconsistency of this study with other surveys. In vitro studies provide constant conditions for comparing materials and techniques [1][2][14][21][22]. This study showed that all sealers allowed apical and coronal leakage; but this will not necessarily cause endodontic failure [23].

## CONCLUSION

None of the tested materials showed superiority in promoting apical and coronal sealing. Finding materials that promote optimal seal to the root canal system are still needed.
